# Gut Microbiome and Kidney Disease in Pediatrics: Does Connection Exist?

**DOI:** 10.3389/fmicb.2016.00235

**Published:** 2016-03-03

**Authors:** Tetyana L. Vasylyeva, Ruchi Singh

**Affiliations:** Department of Pediatrics, Texas Tech University Health Sciences Center, AmarilloTX, USA

**Keywords:** gut microbiome, pediatrics, gut renal axis, inflammatory markers, kidney disease

## Abstract

Child development is a unique and continuous process that is impacted by genetics and environmental factors. Gut microbiome changes with development and depends on the stage of gut maturation, nutrition, and overall health. In spite of emerging data and active study in adults, the gut-renal axis in pediatrics has not been well considered and investigated. This review will focus on the current knowledge of gut microbiota impacts on kidney disease with extrapolation to the pediatric population.

## Introduction

### Summary of Microbial Activities in Human Metabolism

Knowledge of the microbial communities living in human body is increasing at a fast pace. The Human Microbiome Project demonstrated that humans might be considered superorganisms, composed of human and microbial components, interacting in a symbiotic manner (Turnbaugh et al., 2007). Microbial population has coexisted with humans in a state of mutually beneficial cohabitation and plays an important role in health and disease (Cani and Delzenne, 2009). Inter-organismal crosstalk interruption leads to profound and diverse cellular and metabolic changes observed in gut dysbiosis (Hooper and Gordon, 2001). Gordon et al. showed that the composition of the gut microbiome affects metabolism and energy homeostasis (Ley et al., 2007; Turnbaugh et al., 2007). The gut microbiome is a biotic factor regulating body weight, and thus linked to obesity and other metabolic disorders (Ley et al., 2006) and (Cani and Delzenne, 2009). Maintaining the symbiotic equilibrium between gut microbiome and host in different pathological conditions remains a challenge for the physicians. The understanding of their interaction, manipulation of the structure and functions of human microbiota may allow effective prevention and treatment of many diseases.

### Small Intestinal Bacterial Overgrowth may Triggers CKD/ESRD

Dysbiosis can be promoted by any inflammatory reaction, making it difficult to discern cause and consequence of the disease (Sansonetti, 2008; Anders et al., 2013). The gut microbiome could be a trigger for the deregulated immune system in kidney disease (Anders et al., 2013).

Anders et al. (2013) pointed out that the metabolic alterations of uremia favor gut pathogen overgrowth. But this fact has been largely neglected as a trigger for chronic kidney disease (CKD)/end-stage renal disease (ESRD)-related immune derangements (Martin et al., 2009). The concept was that pathobiont overgrowth induces inflammation and loss of barrier function that, results in increased translocation of bacterial components such as LPS and other immune stimulating proteins. This process activates innate immunity characterized by production of pro-inflammatory cytokines and modulates a number of clinically relevant processes in CKD such as the progression of CKD, accelerated atherogenesis, and protein wasting (Anders et al., 2013). Data from a previous studies indicated that toxic products generated by a dysbiotic gut microbiome may contribute to progression of CKD and CKD-related complications (Stecher et al., 2007; Wu et al., 2011; Ramezani and Raj, 2014). As a result, a hypothesis was developed that probiotics and prebiotics reduce progression of CKD and associated uremia by optimizing the gut microbiome (Lin et al., 2011).

With a comprehensive understanding of the structure, density, and function of the gut microbiota, new therapeutic targets could be identified and utilized for a healthier gut. A healthy gut could help improve overall well-being (Vitetta and Gobe, 2013) including health improvement for children with acute and chronic renal conditions.

During development, human microbiome stimulates and synchronizes with the host innate immune functions by the classical complement pathway, the alternate pathway, and the mannose-binding lectin pathway. Kidney disease such as atypical hemolytic uremic syndrome (aHUS) and membranoproliferative glomerulonephritis are closely linked to abnormalities in complement activation due to host genetic anomalies (Sugimoto et al., 2012). Animal experiments have also established that the microbiota modulate the expression of host genes (Bäckhed et al., 2004). Moreover gnotobiotic mouse models revealed that different members of the microbiota induce different types of immune responses (Ivanov et al., 2009). A mixture of *Clostridium* sp. demonstrated induction of FoxP3+ regulatory T cells and the segmented filamentous bacteria promoted T helper type 17 cell differentiation (Soulage et al., 2013). Animal models have advanced our understanding of the role of microflora in human disease. However, animal conditions can only mimic the features of human disorders (Nagpal et al., 2014). The gut microbiota produces uremic toxins such as advanced glycation end-products, phenols [e.g., *p*-cresyl sulfate (PCS)], and indoles [e.g., indoxyl sulfate (IS); Bone et al., 1976; Tlaskalová-Hogenová et al., 2011; Hegab et al., 2012]. Animal studies have shown that the biological effect of these molecules include induction of pro-inflammatory responses, leukocyte stimulation, and endothelial dysfunction. These conditions play a substantial role in the development and progression of multiple causes of acute kidney injury (AKI) and CKD (Charney et al., 1993; Motojima et al., 2003; Schepers et al., 2007; Macfarlane and Macfarlane, 2012). IS and PCS are renal and cardiovascular toxins, produced solely by the gut microbiota, which have pro-inflammatory and pro-oxidative properties (Dou et al., 2004). Animal studies have shown that uremic toxins may promote the progression of chronic renal failure by damaging tubular cells (Rossi et al., 2014).

Many reports showed that the composition of gut microbiota is different in CKD patients. Vaziri et al. (2012), using a rat model, showed CKD alters the composition of intestinal microbial flora (Claus et al., 2008). The study revealed substantially less species richness as measured by the number of operational taxonomic units (OTUs) in the nephrectomized rats compared with the controls (Vaziri et al., 2012). The mechanism of bacterial gut alteration was thought to be the result of multiple factors including, but not limited to, metabolic acidosis, volume overload, intestinal wall congestion retention, uremia, frequent use of antibiotics, oral iron, and intestinal ischemia (Kang, 1993; Maslowski and Mackay, 2011; Vaziri et al., 2012). Animal experiments also showed that persistent inflammation contributed to the high rates of CKD development (Vaziri et al., 1985; Stenvinkel et al., 1999).

Some thought that a healthy high fiber diet might promote significant colonization with Shiga toxins-producing *Escherichia coli*, which can lead to hemolytic-uremic syndrome (HUS; Ivanov et al., 2009). Interesting work by Eaton et al. (2011), showed that the probiotic *Lactobacillus reuteri* (ATCC PTA 6475) was effective in suppressing disease symptoms of HUS. Their data indicated that *L. reuteri* partially protected mice from disease manifestations (Eaton et al., 2011). The progression of CKD was hindered by ‘enteric dialysis’ by administering non-pathogenic soil-borne alkalophilic urease-positive bacterium *Sporosarcina pasteurii* (Sp) in 5/6 nephrectomized rats. That pilot study demonstrated that rats fed with 10^9^ cfu/day of live Sp had reduced blood urea-nitrogen levels and showed significantly prolonged lifespans (Mandal et al., 2013). Furthermore, in germ-free mice that are lacking in immune related to inflammation, the gut microbiome was found to influence kidney homeostasis with elevated levels of key cell volume regulators (betaine, choline, and myo-inositol) in kidneys (Ranganathan et al., 2006).

Obesity and metabolic disorders link tightly to kidney disease. Studies in mice demonstrated that an interrelationship exists between energy balance, diet, and the composition of the gut microbial community. This interrelationship might have significant clinical implications including obesity related complications (Bäckhed et al., 2004, 2007; Ley et al., 2005; Turnbaugh et al., 2006; Clavel and Haller, 2007). In a murine model, gut microbiome product PCS interfered with intracellular insulin signaling pathways and triggered insulin resistance. The treatment of CKD mice with a prebiotic reduced the intestinal production and blood levels of PCS and prevented insulin resistance and lipid abnormalities (Turnbaugh et al., 2008).

To improve animal models to study the human microbiome, a gnotobiotic piglet model was developed (Wang and Donovan, 2015) furthermore human microbiota-associated (HMA) piglets have been established using inocula from infants, children, and adults. The gut microbiota of recipient HMA piglets was more similar to that of the human donor than that of conventionally reared piglets harboring a pig microbiota. Moreover, *Bifidobacterium* and *Bacteroides*, two predominant bacterial groups of the infant gut, had been successfully established in the HMA piglets.

## What did we Learn About Gut Microbiome and Renal Function From Animal Studies?

Understanding impact of gut microbiome and pathobiome in renal pathology might bring a critical new knowledge in understanding genesis and treatment of kidney disease. The metagenomics approach has the potential to uncover entirely novel genes, gene families, and their encoded proteins, which might be of biotechnological and pharmaceutical relevance (Belizario and Napolitano, 2015). Therefore, animal models strongly proved impact on gut microbiome and renal function, in addition suitable animal models were created to pursue relevant study in pediatric population.

## Gut Microbiome and Renal Axis in Adult Population

An adult human’s microbiome is diverse and dependent upon health status, diet, and geographic location. The gut harbors a complex mixture of tens of trillions of microbes, comprised of more than 1,000 diverse species of identified bacteria with over three million genes. In healthy individuals, the phyla Bacteroidetes and Firmicutes contribute >90% of all species, including abundant bacterial genera such as *Bacteroides* sp., *Alistipes* sp., *Prevotella* sp., *Porphyromonas* sp., *Clostridium* sp., *Dorea* sp., *Faecalibacterium* sp., *Eubacterium* sp., *Ruminococcus* sp., and *Lactobacillus* sp. Other less abundant phyla include the Actinobacteria (that is, *Bifidobacterium* sp. and *Collinsella* sp.), Proteobacteria (that is, *Enterobacteriaceae, Sutterella* sp., and *Helicobacter* sp.), Verrucomicrobia (that is, *Akkermansia* sp.), and some others (Qin et al., 2010).

The microbiota colonizing the gut control the normal development and function of the mucosal barriers, support food digestion, and defend the individual from circulation of pathogenic micro-organisms. On the other hand, the break down of proteins and peptides by colonic microorganisms yields a great diversity of end-products, many of which have toxic properties (Mafra et al., 2013).

Adult uremic patients show greatly increased counts of both aerobic and anaerobic organisms in the duodenum and jejunum, portions of the intestines not normally colonized heavily by bacteria in healthy persons (Ramezani and Raj, 2014). With diminished kidney clearance, quantitative, and qualitative alterations in gut microbiota were noted in adult patients with CKD and ESRD (Hida et al., 1996; Simenhoff et al., 1996; Vaziri et al., 2013). Vaziri et al. (2013) showed significant differences in the abundance of 190 microbial OTUs between the patients with ESRD and the normal control individuals. In addition, hemodialysis patients had significantly lower numbers of *Bifidobacteria* and higher *Clostridium perfringens* numbers (Rossi et al., 2014).

Intestinal microbial flora in patients with CKD characterized by decreases in both *Lactobacillaceae* and *Prevotellaceae* families have been reported (Mafra et al., 2014). As a result, uremic toxins produce bacteria families’ over growth that impacts immune response and inflammatory reactions. Study in 149 CKD patients with a mean estimated Glomerular Filtration Rate (eGFR) of 40 ± 9 mL/min/1.73 m^2^ showed that serum free and total IS were independently associated with increased levels of serum IL-6, TNF-α, and IFN-γ, whereas serum free and total PCS were independently associated with increased levels of serum IL-6 and pulse wave velocity (Rossi et al., 2014). This study for the first time proved a strong connection between produced and retained uremic toxins and demonstrated strong predictors of cardiovascular mortality in CKD patients. The idea was further supported by a publication of Mafra et al. (2014). They also provided a concise description of the potential role of the CKD-associated changes in the gut microbiome and its potential role in the pathogenesis of inflammation and uremic toxicity (Mafra et al., 2014). PCS also contributed to the development of insulin resistance in patients with CKD (Soulage et al., 2013).

Lipopolysaccharide (LPS)-induced monocyte/macrophage activation could explain systemic inflammation and might be relevant to CKD/ESRD patients’ conditions (Stearns-Kurosawa et al., 2011). Cani et al. (2007) demonstrated in mouse model that the metabolic endotoxemia dysregulates the inflammatory character and activates body weight gain and diabetes. They also observed that lowering plasma LPS concentration could be an effective approach to regulate metabolic diseases such as diabetes (Cani et al., 2007).

We recently reported significant increases in inflammatory markers and markers of endothelial dysfunction in patients with diabetes, CKD, and ESRD on peritoneal dialysis, as well as correlations with levels of LPS and zonulin (a gut permeability marker; Chennasamudram et al., 2013; Nakhla et al., 2014; Singh et al., 2014). Human intestine also acts as an active player by presenting more precursors for fermentation due to disturbances in assimilation caused by uremia, followed by alterations in further processing related to changes in the composition of the fermenting flora (Schepers et al., 2010). Deficiency in vitamin K and calcidiol is a common feature among patients with CKD and ESRD and is likely attributed to gut dysbiosis (LaClair et al., 2005; Pilkey et al., 2007).

The colonic microbiome is an active site of methanol production, which might appear in the exhaled breath of subjects. The study of methanol breath content in ESRD on hemodialysis showed that methanol production could be substantially manipulated by the diet, which changed gut populations *per se* (Lee et al., 2012*)*.

Strong connection between gut pathobionts and CKD and ESRD in adult population is nowadays undoubtful fact. The next important questions to be answered in nephrology: how gut microbiome diversity impacted by primary renal disease and how it changes with progression of chronic renal impairment.

## Pediatric Gut Microbiome

**FIGURE 1:**
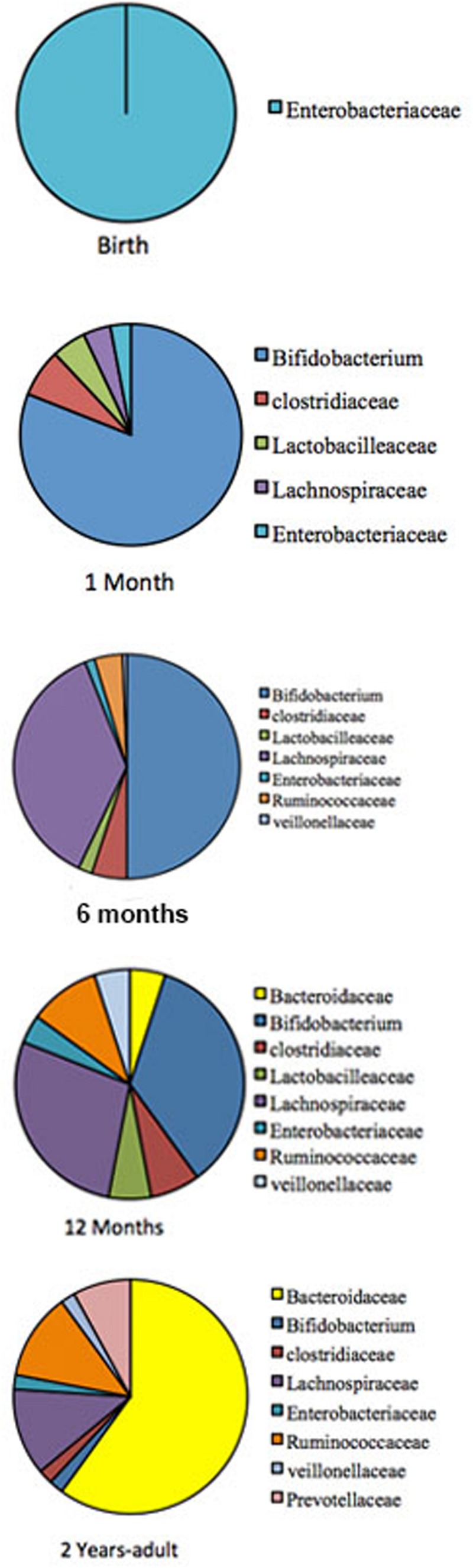
**The pattern of GIT microbiome development during a child development.** Pie charts are showing profusion of the bacterial taxa at different development stage. The gut-microbiome of the new born is primarily colonized by *Enterobacteria* (Gosalbes et al., 2013). During the first month, *Bifidobacterial species* pre-dominate in the gut (Koenig et al., 2011), but at around 4–6 months is convoyed by an increase of clostridial species. Later, the microbiome composition consists of mainly Bacteroidaceae, Lachnospiraceae, and Ruminococcaceae, which then remains stable into adulthood (Fanaro et al., 2005; Morelli, 2008; Lozupone et al., 2012).

The gastrointestinal tract (GIT) in neonates was thought to be almost sterile at birth and then rapidly colonized by bacteria shortly after delivery until an adult type composition was acquired around the first to the third year of life (Rotimi and Duerden, 1981; Valle et al., 2007; DiGiulio et al., 2008; O’Toole and Claesson, 2010). This view has recently been challenged by reports of detection of diverse microbes in placenta, umbilical cord, amniotic fluid, and meconium (Jiménez et al., 2005, 2008; Dominguez-Bello et al., 2010; Vallés et al., 2012; Gosalbes et al., 2013; Moles et al., 2013; Song et al., 2013; Aagaard et al., 2014). It is now well established that during the first 3 years of life, children experience significant developmental changes that influence their health status as well as their immune system. Using sequential fecal analysis in a large cohort, human microbiome studies across North America, Africa, South America, and Europe, have made it apparent that gut microbiome is highly unstable during the first 3 years of life (De Filippo et al., 2010; Koenig et al., 2011; Yatsunenko et al., 2012). The pattern of GIT microbiome development during child development is summarized in **Figure 1**.

## Gut Microbiome in Children with Kidney Disease

**FIGURE 2:**
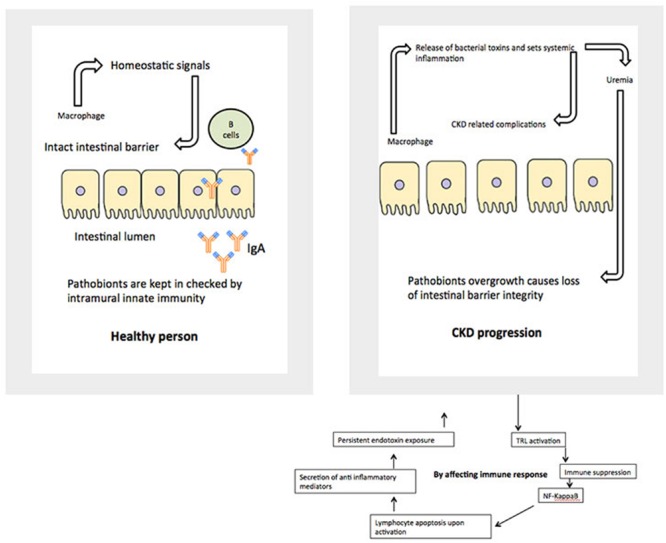
**Potential mechanisms by which gut microbiome affects kidney functions**.

The relationship between alterations in the gut microbiome and its possible involvement in the development of renal disease later in life is an important venue to be investigated. Consideration of GIT barrier structure and it changes in pathological conditions is necessary to understand relationships in the gut-renal axis in pediatrics. The maintenance of a healthy intestinal barrier is extremely important in children. There are multiple layers, which makeup the barrier between gut lumen and the rest of the body. The physical barrier is composed of gut microbiota, mucus, epithelial cells, and the innate and adaptive immune cells forming the gut-associated lymphoid tissue. The internal mucosal layer is dense and does not allow bacteria to penetrate, while the external mucus layer is the habitat of the gut microbiota. Each of those layers is compromised in a uremic condition (**Figure 2**).

As mentioned above, in a healthy body, normal gut microbiota competes with pathogens for space and energy resources, processes the molecules necessary to maintain mucosal integrity, and modulate the immunological activity of the deep barrier. In CKD and ESRD these functions are greatly compromised, based on animal and adult patient studies. Children with similar renal conditions are usually on a very specific diet, which might include food low in potassium, phosphorus, and salt. They may use phosphate binders and have restrictions on their water consumption. During infancy and early childhood, these restrictions could contribute to the development of an abnormal microbial population and weaken the first line of the gut barrier (Vaziri et al., 2013). This eventually leads to a “leaky gut syndrome.” It also further compromises vitamin absorption necessary for growing bodies, including Vitamin D.

We also presume that many drugs or compounds used in the treatment of CKD and ESRD not only compromise gut microbiome, but may experience abnormal pharmacokinetics in the setting of abnormal intestinal permeability. In addition, the development of new pharmacological approaches to modulate the gut barrier components might contribute to prevention and treatment of extra-intestinal diseases, including kidney pathology.

Does early-life dysbiosis precede and play a role in disease pathogenesis, or simply originate because of the disease process itself? This question has been answered in a few diseases, including inflammatory bowel disease, obesity, and asthma (Arrieta et al., 2014). A paucity of data exists regarding how the gut-renal axis functions in a growing human with renal disease and what the role of the microbiome is in this process. Although, some data gathered from bench research and knowledge obtained from adult populations might be extrapolated to the pediatric population, the unique growing and constantly changing body deserves separate investigation.

One of the challenges in the study of the microbiome in any disease, including kidney disease, in early childhood is the greater variation in strain diversity documented during the first few months and years of life (Viggiano et al., 2015). Gut microbiota evolve and change during that growth. Data showed that individuals harbor their own specific *Bifidobacterial* microbiota even at the strain level. Thus, 96% of infants possessed their own individual specific *Bifidobacterial* microbiota (Barrett et al., 2015).

Study of gut microbiome in children with renal disease should focus on four types of dysbiosis: loss of keystone taxa, loss of diversity, shifts in metabolic capacity, and blooms of pathogens. Both genetic and environmental factors should be taken into consideration while studying the development of gut microbiota composition (Spor et al., 2011). For example, one of the relevant papers from China found that gut microbiota was involved in melamine-induced renal injury in infants and children exposed to melamine-tainted milk. Melamine-induced renal toxicity was found to be mediated by the gut microbiota (Zheng et al., 2013).

Preterm children need special consideration relevant to renal pathology. It is known that children born preterm have a high risk of the fast progression of CKD and low nephron numbers (Kandasamy et al., 2012; Brennan and Kandasamy, 2013; Carmody and Charlton, 2013; Mishra et al., 2014). Many infants begin life with a significant number of immature nephrons. They are exposed to a variety of external stressors such as hemodynamic alterations, nephrotoxic medications, infections, and suboptimal nutrition that can delay ongoing kidney development or cause nephron loss. Preterm infants and particularly very low birth weight infants are also at a disadvantage when it comes to the development of a healthy microbiome (Gritz and Bhandari, 2015). The causes of their gut immaturity include preterm rupture of membranes, maternal infection, Cesarean delivery, perinatal and postnatal broad-spectrum antibiotic exposure, exposure to other gut-modifying medications (which might alter gut permeability), periods of fasting, intensive care infection control standards and exposure to resistant microbes and decreased acquaintance of human milk (Scholtens et al., 2012; Berrington et al., 2013; Gupta et al., 2013). Arboleya et al. (2012) compared full-term breastfed vaginally delivered infants with preterm infants with regard to differences in representation of 18 microbial groups within gut flora. They demonstrated that when compared with full-term infants, preterm infants showed increased populations of facultative anaerobes such as *Enterococcus, Enterobacter*, and *Lactobacillus*, increased numbers of *Staphylococcus*, and decreased numbers of anaerobes such as *Bifidobacterium, Bacteroides*, and *Atopobium* (Madan et al., 2012; Berrington et al., 2014).

The gut microbiome specifics in relationship to kidney compromised status were not well investigated.

Cross-sectional analysis of pediatric CKD data has revealed valuable information that better defines the prevalence of comorbid conditions and associated risk factors, but not gut microbiome. Hypertension, left ventricular hypertrophy, dyslipidemia, anemia, poor growth, and abnormal neurocognitive development are known comorbidities that accompany CKD (Wong et al., 2012), but pediatric gut microbiome in a uremic milieu is yet to be evaluated.

## Conclusion

Extensive microbiome research has been made possible by recent advances in gene sequencing and bioinformatics tools. Further study is required to decipher structures and functions of microbe population in the gut of pediatric AKI, CKD, and ESRD patients. This is a call for concerted efforts of pediatricians, pediatric nephrologists, immunologists, microbiologists, food and nutrition experts, and computational biologists to understand this clinical phenomenon and further applications in developing therapeutic regimens.

## Author Contributions

TV and RS contributed to the conception, design of review. TV has drafted the work critically for important intellectual content. TV has provided final approval of the version to be published. Authors have agreed to be accountable for all aspects of the work in ensuring that questions related to the accuracy or integrity of any part of the work are appropriately investigated and resolved.

## Conflict of Interest Statement

The authors declare that the research was conducted in the absence of any commercial or financial relationships that could be construed as a potential conflict of interest.

## References

[B1] Aagaard K. Ma J. Antony K. M. Ganu R. Petrosino J. Versalovic J. (2014). The placenta harbors a unique microbiome. *Sci. Transplant. Med.* 6:237ra65 10.1126/scitranslmed.3008599 PMC4929217 24848255

[B2] Anders H. J. Andersen K. Stecher B. (2013). The intestinal microbiota, a leaky gut, and abnormal immunity in kidney disease. *Kidney Int.* 83 1010–1016. 10.1038/ki.2012.440 23325079

[B3] Arboleya S. Binetti A. Salazar N. Fernández N. Solís G. Hernández-Barranco A. (2012). Establishment and development of intestinal microbiota in preterm neonates. *FEMS Microbiol. Ecol.* 79 763–772. 10.1111/j.1574-6941.2011.01261.x 22126419

[B4] Arrieta M. C. Stiemsma L. T. Amenyogbe N. Brown E. M. Finlay B. (2014). The intestinal microbiome in early life: health and disease. *Front. Immunol.* 5:247 10.3389/fimmu.2014.00427 PMC4155789 25250028

[B5] Bäckhed F. Ding H. Wang T. Hooper L. V. Koh G. Y. Nagy A. (2004). The gut microbiota as an environmental factor that regulates fat storage. *Proc. Natl. Acad. Sci. U. S.A.* 101 15718–15723. 10.1073/pnas.0407076101 15505215 PMC524219

[B6] Bäckhed F. Manchester J. K. Semenkovich C. F. Gordon J. I. (2007). Mechanisms underlying the resistance to diet-induced obesity in germ-free mice. *Proc. Natl. Acad. Sci. U. S.A.* 104 979–984. 10.1073/pnas.0605374104 17210919 PMC1764762

[B7] Barrett E. Deshpandey A. K. Ryan C. A. Dempsey E. M. Murphy B. O’Sullivan L. (2015). The neonatal gut harbours distinct bifidobacterial strains. *Arch. Dis. Child. Fetal Neonatal Ed.* 100 F405–F410. 10.1136/archdischild-2014-306110 25896967

[B8] Belizario J. E. Napolitano M. (2015). Human microbiomes and their roles in dysbiosis, common diseases, and novel therapeutic approaches. *Front. Microbiol.* 6:1050 10.3389/fmicb.2015.01050 PMC4594012 26500616

[B9] Berrington J. E. Stewart C. J. Cummings S. P. Embleton N. D. (2014). The neonatal bowel microbiome in health and infection. *Curr. Opin. Infect. Dis.* 27 236–243. 10.1097/QCO.0000000000000061 24751892

[B10] Berrington J. E. Stewart C. J. Embleton N. D. Cummings S. P. (2013). Gut microbiota in preterm infants: assessment and relevance to health and disease. *Arch. Dis. Child. Fetal Neonatal Ed.* 98 F286–F290. 10.1136/archdischild-2012-302134 23009761

[B11] Bone E. Tamm A. Hill M. (1976). The production of urinary phenols by gut bacteria and their possible role in the causation of large bowel cancer. *Am. J. Clin. Nutr.* 29 1448–1454. 826152 10.1093/ajcn/29.12.1448

[B12] Brennan S. Kandasamy Y. (2013). Renal parenchymal thickness as a measure of renal growth in low-birth-weight infants versus normal-birth-weight infants. *Ultrasound Med. Biol.* 39 2315–2320. 10.1016/j.ultrasmedbio.2013.07.001 24035629

[B13] Cani P. D. Amar J. Iglesias M. A. Poggi M. Knauf C. Bastelica D. (2007). Metabolic endotoxemia initiates obesity and insulin resistance. *Diabetes* 56 1761–1772. 10.2337/db06-1491 17456850

[B14] Cani P. D. Delzenne N. M. (2009). The role of the gut microbiota in energy metabolism and metabolic disease. *Curr. Pharm. Des.* 15 1546–1558. 10.2174/138161209788168164 19442172

[B15] Carmody J. B. Charlton J. R. (2013). Short-term gestation, long-term risk: prematurity and chronic kidney disease. *Pediatrics* 131 1168–1179. 10.1542/peds.2013-0009 23669525

[B16] Charney D. I. Walton D. F. Cheung A. K. (1993). Atherosclerosis in chronic renal failure. *Curr. Opin. Nephrol. Hypertens* 2 876–882. 10.1097/00041552-199311000-00004 7922227

[B17] Chennasamudram S. P. Noor T. Vasylyeva T. L. (2013). Comparison of sevelamer and calcium carbonate on endothelial function and inflammation in patients on peritoneal dialysis. *J. Ren. Care* 39 82–89. 10.1111/j.1755-6686.2013.12009.x 23574727

[B18] Claus S. P. Tsang T. M. Wang Y. Cloarec O. Skordi E. Martin F. P. (2008). Systemic multicompartmental effects of the gut microbiome on mouse metabolic phenotypes. *Mol. Syst. Biol.* 4:219 10.1038/msb.2008.56 PMC2583082 18854818

[B19] Clavel T. Haller D. (2007). Molecular interactions between bacteria, the epithelium, and the mucosal immune system in the intestinal tract: implications for chronic inflammation. *Curr. Issues Intest. Microbiol.* 8 25–43. 17542334

[B20] De Filippo C Cavalieri D. Di Paola M. Ramazzotti M. Poullet J. B. Massart S. (2010). Impact of diet in shaping gut microbiota revealed by a comparative study in children from Europe and rural Africa. *Proc. Natl. Acad. Sci. U.S.A.* 107 14691–14696. 10.1073/pnas.1005963107 20679230 PMC2930426

[B21] DiGiulio D. B. Romero R. Amogan H. P. Kusanovic J. P. Bik E. M. Gotsch F. (2008). Microbial prevalence, diversity and abundance in amniotic fluid during preterm labor: a molecular and culture-based investigation. *PLoS ONE* 3:e3056 10.1371/journal.pone.0003056 PMC2516597 18725970

[B22] Dominguez-Bello M. G. Costello E. K. Contreras M. Magris M. Hidalgo G. Fierer N. (2010). Delivery mode shapes the acquisition and structure of the initial microbiota across multiple body habitats in newborns. *Proc. Natl. Acad. Sci. U.S.A.* 107 11971–11975. 10.1073/pnas.1002601107 20566857 PMC2900693

[B23] Dou L. Bertrand E. Cerini C. Faure V. Sampol J. Vanholder R. (2004). The uremic solutes p-cresol and indoxylsulfate inhibit endothelial proliferation and wound repair. *Kidney Int.* 65 442–451. 10.1111/j.1523-1755.2004.00399.x 14717914

[B24] Eaton K. A. Honkala A. Auchtung T. A. Britton R. A. (2011). Probiotic *Lactobacillus reuteri* ameliorates disease due to enterohemorrhagic *Escherichia coli* in germfree mice. *Infect. Immun.* 79 185–191. 10.1128/IAI.00880-10 20974822 PMC3019869

[B25] Fanaro S. Boehm G. Garssen J. Knol J. Mosca F. Stahl B. (2005). Galacto-oligosaccharides and long-chain fructo-oligosaccharides as prebiotics in infant formulas: a review. *Acta Paediatr. Suppl.* 94 22–26. 16214761 10.1111/j.1651-2227.2005.tb02150.x

[B26] Gosalbes M. J. Llop S. Vallès Y. Moya A. Ballester F. Francino M. P. (2013). Meconium microbiota types dominated by lactic acid or enteric bacteria are differentially associated with maternal eczema and respiratory problems in infants. *Clin. Exp. Allergy* 43 198–211. 10.1111/cea.12063 23331561

[B27] Gritz E. C. Bhandari V. (2015). The human neonatal gut microbiome: a brief review. *Front. Pediatr.* 3:17 10.3389/fped.2015.00060 PMC4350424 25798435

[B28] Gupta R. W. Tran L. Norori J. Ferris M. J. Eren A. M. Taylor C. M. (2013). Histamine-2 receptor blockers alter the fecal microbiota in premature infants. *J. Pediatr. Gastroenterol. Nutr.* 56 397–400. 10.1097/MPG.0b013e318282a8c2 23254444

[B29] Hegab Z. Gibbons S. Neyses L. Mamas M. A. (2012). Role of advanced glycation end products in cardiovascular disease. *World J. Cardiol.* 4 90–102. 10.4330/wjc.v4.i4.90 22558488 PMC3342583

[B30] Hida M. Aiba Y. Sawamura S. Suzuki N. Satoh T. Koga Y. (1996). Inhibition of the accumulation of uremic toxins in the blood and their precursors in the feces after oral administration of Lebenin, a lactic acid bacteria preparation, to uremic patients undergoing hemodialysis. *Nephron* 74 349–355. 10.1159/000189334 8893154

[B31] Hooper L. V. Gordon J. I. (2001). Commensal host-bacterial relationships in the gut. *Science* 292 1115–1118. 10.1126/science.1058709 11352068

[B32] Ivanov I. I. Atarashi K. Manel N. Brodie E. L. Shima T. Karaoz U. (2009). Induction of intestinal Th17 cells by segmented filamentous bacteria. *Cell* 139 485–498. 10.1016/j.cell.2009.09.033 19836068 PMC2796826

[B33] Jiménez E. Fernández L. Marín M. L. Martín R. Odriozola J. M. Nueno-Palop C. (2005). Isolation of commensal bacteria from umbilical cord blood of healthy neonates born by cesarean section. *Curr. Microbiol.* 51 270–274. 10.1007/s00284-005-0020-3 16187156

[B34] Jiménez E. Marín M. L. Martín R. Odriozola J. M. Olivares M. Xaus J. (2008). Is meconium from healthy newborns actually sterile? *Res. Microbiol.* 159 187–193. 10.1016/j.resmic.2007.12.007 18281199

[B35] Kandasamy Y. Smith R. Wright I. M. R. (2012). Oligonephropathy of prematurity. *Am. J. Perinatol.* 29 115–120. 10.1055/s-0031-1295651 22094915

[B36] Kang J. Y. (1993). The gastrointestinal tract in uremia. *Dig. Dis. Sci.* 38 257–268. 10.1007/BF01307542 8425438

[B37] Koenig J. E. Spor A. Scalfone N. Fricker A. D. Stombaugh J. Knight R. (2011). Succession of microbial consortia in the developing infant gut microbiome. *Proc. Natl. Acad. Sci. U.S.A.* 108 4578–4585. 10.1073/pnas.1000081107 20668239 PMC3063592

[B38] LaClair R. E. Hellman R. N. Karp S. L. Kraus M. Ofner S. Li Q. (2005). Prevalence of calcidiol deficiency in CKD: a cross-sectional study across latitudes in the United States. *Am. J. Kidney Dis.* 45 1026–1033. 10.1053/j.ajkd.2005.02.029 15957131

[B39] Lee H. J. Pahl M. V. Vaziri N. D. Blake D. R. (2012). Effect of hemodialysis and diet on the exhaled breath methanol concentration in patients with ESRD. *J. Ren. Nutr.* 22 357–364. 10.1053/j.jrn.2011.07.003 22100775

[B40] Ley R. E. Bäckhed F. Turnbaugh P. Lozupone C. A. Knight R. D. Gordon J. I. (2005). Obesity alters gut microbial ecology. *Proc. Natl. Acad. Sci. U.S.A.* 102 11070–11075. 10.1073/pnas.0504978102 16033867 PMC1176910

[B41] Ley R. E. Knight R. Gordon J. I. (2007). The human microbiome: eliminating the biomedical/environmental dichotomy in microbial ecology. *Environ. Microbiol.* 9 3–4. 10.1111/j.1462-2920.2006.01222_3.x 17227400

[B42] Ley R. E. Turnbaugh P. J. Klein S. Gordon J. I. (2006). Microbial ecology: human gut microbes associated with obesity. *Nature* 444 1022–1023. 10.1038/4441022a 17183309

[B43] Lin C. J. Chen H. H. Pan C. F. Chuang C. K. Wang T. J. Sun F. J. (2011). p-Cresylsulfate and indoxyl sulfate level at different stages of chronic kidney disease. *J. Clin. Labor. Anal.* 25 191–197. 10.1002/jcla.20456 PMC6647585 21567467

[B44] Lozupone C. A. Stombaugh J. I. Gordon J. I. Jansson J. K. Knight R. (2012). Diversity, stability and resilience of the human gut microbiota. *Nature* 489 220–30. 10.1038/nature11550 22972295 PMC3577372

[B45] Macfarlane G. T. Macfarlane S. (2012). Bacteria, colonic fermentation, and gastrointestinal health. *J. AOAC Int.* 95 50–60. 10.5740/jaoacint.SGE_Macfarlane 22468341

[B46] Madan J. C. Farzan S. F. Hibberd P. L. Karagas M. R. (2012). Normal neonatal microbiome variation in relation to environmental factors, infection and allergy. *Curr. Opin. Pediatr.* 24 753–759. 10.1097/MOP.0b013e32835a1ac8 23111681 PMC3914299

[B47] Mafra D. Barros A. F. Fouque D. (2013). Dietary protein metabolism by gut microbiota and its consequences for chronic kidney disease patients. *Future Microbiol.* 8 1317–1323. 10.2217/fmb.13.103 24059921

[B48] Mafra D. Lobo J. C. Barros A. F. Koppe L. Vaziri N. D. Fouque D. (2014). Role of altered intestinal microbiota in systemic inflammation and cardiovascular disease in chronic kidney disease. *Future Microbiol.* 9 399–410. 10.2217/fmb.13.165 24762311

[B49] Mandal A. Das K. Roy S. Mondal KCh Nandi D. K. (2013). In vivo assessment of bacteriotherapy on acetaminophen-induced uremic rats. *J. Nephrol.* 26 228–236. 10.5301/jn.5000129 22782327

[B50] Martin F. P. Sprenger N. Yap I. K. Wang Y. Bibiloni R. Rochat F. (2009). Panorganismal gut microbiome–host metabolic crosstalk. *J. Proteome Res.* 8 2090–2105. 10.1021/pr801068x 19281268

[B51] Maslowski K. M. Mackay C. R. (2011). Diet, gut microbiota and immune responses. *Nat. Immunol.* 12 5–9. 10.1038/ni0111-5 21169997

[B52] Mishra K. Datta V. Aarushi A. Kaur Narula M. Iyer R. S. Nangia S. (2014). The association between weight for gestational age and kidney volume: a study in newborns in India. *Iran. J. Pediatr.* 24 93–99. 25793052 PMC4359611

[B53] Moles L. Gómez M. Heilig H. Bustos G. Fuentes S. de Vos W. (2013). Bacterial diversity in meconium of preterm neonates and evolution of their fecal microbiota during the first month of life. *PLoS ONE* 8:e66986 10.1371/journal.pone.0066986 PMC3695978 23840569

[B54] Morelli L. (2008). Postnatal development of intestinal microflora as influenced by infant nutrition. *J. Nutr.* 138 1791S–1795S. 18716188 10.1093/jn/138.9.1791S

[B55] Motojima M. Hosokawa A. Yamato H. Muraki T. Yoshioka T. (2003). Uremic toxins of organic anions up-regulate PAI-1 expression by induction of NF-kappaB and free radical in proximal tubular cells. *Kidney Int.* 63 1671–1680. 10.1046/j.1523-1755.2003.00906.x 12675842

[B56] Nagpal R. Yadav H. Marotta F. (2014). Gut microbiota: the next-gen frontier in preventive and therapeutic medicine? *Front. Med.* 1:15 10.3389/fmed.2014.00015 PMC4341269 25767799

[B57] Nakhla E. Singh R. Chennasamudram S. Vasylyeva O. Naguib T. Vasylyeva T. L. (2014). Sex disparity in cardiovascular mortality in patient with end-stage renal disease and Type 2 diabetes mellitus. *Diabetes Res. Treatm.* 1 111–114.

[B58] O’Toole P. W. Claesson M. J. (2010). Gut microbiota: changes throughout the lifespan from infancy to elderly. *Int. Dairy J.* 20 281–291. 10.1016/j.idairyj.2009.11.010

[B59] Pilkey R. M. Morton A. R. Boffa M. B. Noordhof C. Day A. G. Su Y. (2007). Subclinical vitamin K deficiency in hemodialysis patients. *Am. J. Kidney Dis.* 49 432–439. 10.1053/j.ajkd.2006.11.041 17336705

[B60] Qin J. Li R. Raes J. Arumugam M. Burgdorf K. S. Manichanh C. (2010). A human gut microbial gene catalogue established by metagenomic sequencing. *Nature* 464 59–U70. 10.1038/nature08821 20203603 PMC3779803

[B61] Ramezani A. Raj D. S. (2014). The gut microbiome, kidney disease, and targeted interventions. *J. Ame. Soc. Nephrol.* 25 657–670. 10.1681/ASN.2013080905 PMC3968507 24231662

[B62] Ranganathan N. Patel B. G. Ranganathan P. Marczely J. Dheer R. Pechenyak B. (2006). In vitro and in vivo assessment of intraintestinal bacteriotherapy in chronic kidney disease. *ASAIO J.* 52 70–79. 10.1097/01.mat.0000191345.45735.00 16436893

[B63] Rossi M. Campbell K. L. Johnson D. W. Stanton T. Vesey D. A. Coombes J. S. (2014). Protein-bound uremic toxins, inflammation and oxidative stress: a cross-sectional study in stage 3-4 chronic kidney disease. *Arch. Med. Res.* 45 309–317. 10.1016/j.arcmed.2014.04.002 24751327

[B64] Rotimi V. O. Duerden B. I. (1981). The development of the bacterial flora in normal neonates. *J. Med. Microbiol.* 14 51–62. 10.1099/00222615-14-1-51 7463467

[B65] Sansonetti P. J. (2008). Host-bacteria homeostasis in the healthy and inflamed gut. *Curr. Opin. Gastroenterol.* 24 435–439. 10.1097/MOG.0b013e32830007f7 18622156

[B66] Schepers E. Glorieux G. Vanholder R. (2010). The gut: the forgotten organ in uremia? *Blood Purif.* 29 130–136. 10.1159/000245639 20093818

[B67] Schepers E. Meert N. Glorieux G. Goeman J. Van der Eycken J. Vanholder R. (2007). P-cresylsulphate, the main in vivo metabolite of p-cresol, activates leucocyte free radical production. *Nephrol. Dial. Transplant.* 22 592–596. 10.1093/ndt/gfl584 17040995

[B68] Scholtens P. A. Oozeer R. Martin R. Amor K. B. Knol J. (2012). The early settlers: intestinal microbiology in early life. *Annu. Rev. Food Sci. Technol.* 3 425–447. 10.1146/annurev-food-022811-101120 22224552

[B69] Simenhoff M. L. Dunn S. R. Zollner G. P. Fitzpatrick M. E. Emery S. M. Sandine W. E. (1996). Biomodulation of the toxic and nutritional effects of small bowel bacterial overgrowth in end-stage kidney disease using freeze-dried *Lactobacillus acidophilus*. *Miner. Electrolyte Metab.* 22 92–96. 8676836

[B70] Singh R. Chennasamudram S. P. Sheth S. Vasylyeva T. L. (2014). Correlation of fibroblast growth factor 23 with markers of inflammation and endothelial dysfunction in end-stage renal disease and Type 2 diabetes patients on peritoneal dialysis. *Diab. Metab.* 5 1–5.

[B71] Song S. J. Dominguez-Bello M. G. Knight R. (2013). How delivery mode and feeding can shape the bacterial community in the infant gut. *Can. Med. Assoc. J.* 185 373–374. 10.1503/cmaj.130147 23401408 PMC3602250

[B72] Soulage C. O. Koppe L. Fouque D. (2013). Protein-bound uremic toxins…new targets to prevent insulin resistance and dysmetabolism in patients with chronic kidney disease. *J. Ren. Nutr.* 23 464–466. 10.1053/j.jrn.2013.06.003 23938300

[B73] Spor A. Koren O. Ley R. (2011). Unravelling the effects of the environment and host genotype on the gut microbiome. *Nat. Rev. Microbiol.* 9 279–290. 10.1038/nrmicro2540 21407244

[B74] Stearns-Kurosawa D. J. Osuchowski M. F. Valentine C. Kurosawa S. Remick D. G. (2011). The pathogenesis of sepsis. *Annu. Rev. Pathol.* 6 19–48. 10.1146/annurev-pathol-011110-130327 20887193 PMC3684427

[B75] Stecher B. Robbiani R. Walker A. W. Westendorf A. M. Barthel M. Kremer M. (2007). *Salmonella enterica* serovar typhimurium exploits inflammation to compete with the intestinal microbiota. *PLoS Biol.* 5:2177–2189. 10.1371/journal.pbio.0050244 17760501 PMC1951780

[B76] Stenvinkel P. Heimbürger O. Paultre F. Diczfalusy U. Wang T. Berglund L. (1999). Strong association between malnutrition, inflammation, and atherosclerosis in chronic renal failure. *Kidney Int.* 55 1899–1911. 10.1046/j.1523-1755.1999.00422.x 10231453

[B77] Sugimoto K. Fujita S. Miyazaki K. Okada M. Takemura T. (2012). C3 glomerulonephritis associated with a missense mutation in the factor H gene. *Tohoku J. Exp. Med.* 227 211–215. 10.1620/tjem.227.211 22790979

[B78] Tlaskalová-Hogenová H. Stěpánková R. Kozáková H. Hudcovic T. Vannucci L. Tučková L. (2011). The role of gut microbiota (commensal bacteria) and the mucosal barrier in the pathogenesis of inflammatory and autoimmune diseases and cancer: contribution of germ-free and gnotobiotic animal models of human diseases. *Cell. Mol. Immunol.* 8 110–120. 10.1038/cmi.2010.67 21278760 PMC4003137

[B79] Turnbaugh P. J. Bäckhed F. Fulton L. Gordon J. I. (2008). Diet-induced obesity is linked to marked but reversible alterations in the mouse distal gut microbiome. *Cell Host Microbe* 3 213–223. 10.1016/j.chom.2008.02.015 18407065 PMC3687783

[B80] Turnbaugh P. J. Ley R. E. Hamady M. Fraser-Liggett C. M. Knight R. Gordon J. I. (2007). The human microbiome project. *Nature* 449 804–810. 10.1038/nature06244 17943116 PMC3709439

[B81] Turnbaugh P. J. Ley R. E. Mahowald M. A. Magrini V. Mardis E. R. Gordon J. I. (2006). An obesity-associated gut microbiome with increased capacity for energy harvest. *Nature* 444 1027–1031. 10.1038/nature05414 17183312

[B82] Valle J. M Estepa R. M. Camacho R. M. Estrada R. C. Luna F. G. Guitarte F. B. (2007). Endothelial dysfunction is related to insulin resistance and inflammatory biomarker levels in obese prepubertal children. *Eur. J. Endocrinol.* 156 497–502. 10.1530/EJE-06-0662 17389466

[B83] Vallés Y. Gosalbes M. J. de Vries L. E. Abellán J. J. Francino M. P. (2012). Metagenomics and development of the gut microbiota in infants. *Clin. Microbiol. Infect.* 18 21–26. 10.1111/j.1469-0691.2012.03876.x 22647043

[B84] Vaziri N. D. Dure-Smith B. Miller R. Mirahmadi M. K. (1985). Pathology of gastrointestinal tract in chronic hemodialysis patients: an autopsy study of 78 cases. *Am. J. Gastroenterol.* 80 608–611. 4025276

[B85] Vaziri N. D. Pahl M. V. Crum A. Norris K. (2012). Effect of uremia on structure and function of immune system. *J. Ren. Nutr.* 22 149–156. 10.1053/j.jrn.2011.10.020 22200433 PMC3246616

[B86] Vaziri N. D. Wong J. Pahl M. Piceno Y. M. Yuan J. DeSantis T. Z. (2013). Chronic kidney disease alters intestinal microbial flora. *Kidney Int.* 83 308–315. 10.1038/ki.2012.345 22992469

[B87] Viggiano D. Ianiro G. Vanella G. Bibbò S. Bruno G. Simeone G. (2015). Gut barrier in health and disease: focus on childhood. *Eur. Rev. Med. Pharmacol. Sci.* 19 1077–1085. 25855935

[B88] Vitetta L. Gobe G. (2013). Uremia and chronic kidney disease: the role of the gut microflora and therapies with pro- and prebiotics. *Mol. Nutr. Food Res.* 57 824–832. 10.1002/mnfr.201200714 23450842

[B89] Wang M. Donovan S. M. (2015). Human microbiota-associated swine: current progress and future opportunities. *Ilar J.* 56 63–73. 10.1093/ilar/ilv006 25991699 PMC7108572

[B90] Wong C. J. Moxey-Mims M. Jerry-Fluker J. Warady B. A. Furth S. L. (2012). CKiD (CKD in children) prospective cohort study: a review of current findings. *Am. J. Kidney Dis.* 60 1002–1011. 10.1053/j.ajkd.2012.07.018 23022429 PMC3496011

[B91] Wu I. W. Hsu K. H. Lee C. C. Sun C. Y. Hsu H. J. Tsai C. J. (2011). p-Cresyl sulphate and indoxyl sulphate predict progression of chronic kidney disease. *Nephrol. Dial. Transplant.* 26 938–947. 10.1093/ndt/gfq580 20884620 PMC3042976

[B92] Yatsunenko T. Rey F. E. Manary M. J. Trehan I. Dominguez-Bello M. G. Contreras M. (2012). Human gut microbiome viewed across age and geography. *Nature* 486 222–227. 10.1038/nature11053 22699611 PMC3376388

[B93] Zheng X. J. Zhao A. Xie G. Chi Y. Zhao L. Li H. (2013). Melamine-induced renal toxicity is mediated by the gut microbiota. *Sci. Translat. Med.* 5:172ra22 10.1126/scitranslmed.3005114 23408055

